# Sequential CD38 monoclonal antibody retreatment leads to deep remission in a patient with relapsed/refractory multiple myeloma

**DOI:** 10.1177/2058738420980258

**Published:** 2020-12-23

**Authors:** Maximilian Johannes Steinhardt, Xiang Zhou, Franziska Krummenast, Katharina Meckel, Katharina Nickel, David Böckle, Janin Messerschmidt, Sebastian Knorz, Alexander Dierks, Anke Heidemeier, Constantin Lapa, Hermann Einsele, Leo Rasche, Klaus Martin Kortüm

**Affiliations:** 1 Department of Oncology and Hematology, University Hospital Würzburg, Wurzburg, Germany; 2 Department of Nuclear Medicine, University Hospital Würzburg, Wurzburg, Germany; 3 Department of Nuclear Medicine, University Hospital Augsburg, Augsburg, Germany; 4 Department of Diagnostic Radiology, University Hospital Würzburg, Wurzburg, Germany; 5 Mildred Scheel Early Career Center, University Hospital of Würzburg, Würzburg, Germany

**Keywords:** CD38, MOR202, daratumumab, multiple myeloma, refractory, relapse

## Abstract

We report on a currently 76-year-old female patient with relapsed/refractory (RR) multiple myeloma (MM) treated at our institution. This patient had received six lines of therapy including tandem autologous stem cell transplant, proteasome inhibitor, immunomodulatory drugs and CD38 antibody MOR202. At the last relapse, she progressed during treatment with pomalidomide and MOR202. In an individualized therapy concept, we started a multi-agent salvage therapy with pomalidomide, bortezomib, doxorubicin, dexamethasone, and CD38 antibody daratumumab (“Pom-PAD-Dara”), which resulted in a stringent complete remission with minimal residual disease (MRD) negativity after nine cycles. So far, our patient shows a progression free survival of more than 12 months. Our case demonstrates the feasibility of successful CD38 antibody retreatment in a patient with heavily pretreated CD38 antibody resistant MM.

## Introduction

CD38 is a type II transmembrane glycoprotein, which acts not only as a receptor ruling cell adhesion and signaling events, but also as an ectoenzyme involved in intracellular calcium mobilization.^1^ It has been observed that CD38 is expressed at increased levels on malignant plasma cells in multiple myeloma (MM) and, therefore, CD38 has been regarded as a therapeutic target for MM patients.^2,3^ So far, four monoclonal antibodies targeting CD38, that is, daratumumab, isatuximab, MOR202, and TAK-079 are under clinical investigation or are already approved for the treatment of relapsed/refractory (RR) MM.^4^ Among these four agents, daratumumab is the most widely used in therapy and, recently, it has also been approved for first line combination therapy in newly diagnosed MM patients.^5^ With CD38 directed therapy becoming a key component in the standards of patient care, we increasingly face the problem of drug resistance. However, there is only limited experience with CD38 antibody re-treatment in RRMM patients. Herein, we report a case of RRMM that was successively treated with MOR202- and daratumumab-containing therapies at our institution.

## Case presentation

**Figure 1.**
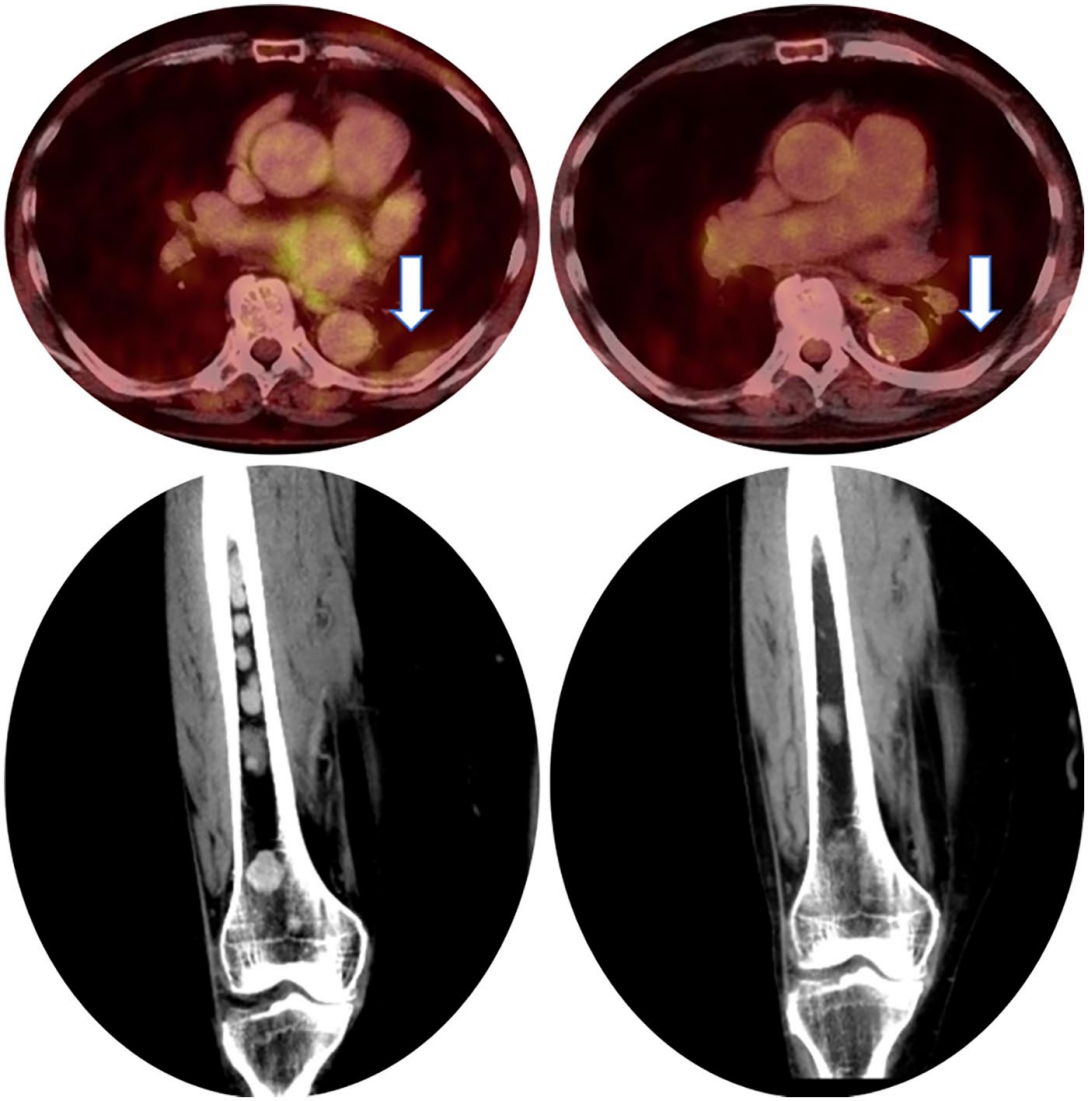
Left: 18F-FDG-PET/CT scan in August 2018 showed pleural extramedullary disease (EMD) and focal bone lesions of the right femur. “Pom-PAD-Dara” (pomalidomide, bortezomib, doxorubicin, dexamethasone, daratumumab) had been initiated 2 days prior to imaging and therefore, we observed a significant reduction of EMD metabolism. Right: 18F-FDG-PET/CT scan in August 2019 demonstrated therapy response after nine cycles of “Pom-PAD-Dara”.

In February 2009, a 65-year old female patient with lower back pain was diagnosed with IgA lambda MM. An M protein level of 19.4 g/l was shown in serum electrophoresis (total Ig A 3000 mg/dl). Bone marrow biopsy revealed a plasma cell infiltration of 40%. Whole body CT scan displayed diffuse bone lesions, and MM induced symptomatic anemia was present. A t(11;14) and a gain1q were found in the Fluorescence in situ Hybridization (FISH) analysis, and an International Staging System (ISS)/Revised International Staging System (R-ISS) stage I was assessed (β2 microglobulin 1.8 mg/l, albumin 4.0 g/dl, lactate dehydrogenase 201 IU/l) resulting in standard risk disease. Following stem cell mobilization with CAD (cyclophosphamide, doxorubicin, dexamethasone) induction, tandem autologous stem cell transplant (ASCT) was performed in March and May 2009, with melphalan given in age-adjusted dosing (140 mg/m²). The patient suffered from grade 3 mucositis, nausea, tachyarrhythmia, and neutropenic fever. Following tandem ASCT and four cycles of experimental bortezomib consolidation within the DSMMX trial, she achieved stringent complete remission (sCR). Nineteen months later (September 2011) the patient lost complete remission (CR), with reappearance of the serum M protein detected by electrophoresis. In February 2012, the M protein level increased to 12 g/l (total Ig A 1450 mg/dl), and the patient again faced a high plasma cell infiltration of 80% in the bone marrow. The patient received lenalidomide and dexamethasone (Rd) relapse therapy within the control arm of the ASPIRE trial. Thirty-nine cycles of Rd could be given until March 2015 with very good partial remission (VGPR) as the best response. In April 2015, disease progressed under treatment, and we switched therapy to bortezomib and dexamethasone (Vd) within the control arm of the MMY3004 CASTOR trial. Unfortunately, no treatment response could be achieved, and the patient progressed after two cycles of Vd. In this situation, the therapy was escalated to experimental “Pom-PAD” (pomalidomide, bortezomib, doxorubicin, dexamethason) combination (“individueller Heilversuch”). She achieved a VGPR after six cycles, before therapy was stopped on the patient’s request. However, only 2 months later, the disease progressed again. 12 cycles of KCyd (carfilzomib, cyclophosphamide, dexamethasone) were administered, and the patient obtained a VGPR as the best response. Remarkably, rapid relapse was observed after the last cycle (M protein 14.1 g/l, Ig A 1980 mg/dl), and the patient was recruited into the MOR201C101 study in June 2017, which tested pomalidomide, dexamethasone and the CD38 antibody MOR202. Overall, she received 13 cycles of this treatment, and VGPR was the best serological response. Of note, under therapy, the patient suffered from serological and extramedullary progression with pleural manifestation (Figure 1). At the same time, she was diagnosed with breast cancer, which could be resected in July 2018. After surgery, we decided for an experimental multi-agent therapy (“individueller Heilversuch”) including pomalidomide, bortezomib, doxorubicin, dexamethasone, and CD38 antibody daratumumab (“Pom-PAD-Dara”).^6^ At therapy start, the M protein level had increased to 7.7 g/l (total Ig A 1446 mg/dl), and bone marrow biopsy revealed an infiltration of 80% of plasma cells. In total, nine cycles of “Pom-PAD-Dara” were administered in this patient. She achieved serological sCR with minimal residual disease (MRD) negativity at a sensitivity level of 10^−5^, and whole body 18F-ﬂuorodeoxyglucose Positron Emission Tomography/Computed Tomography (FDG-PET/CT) scan showed a significant reduction of both medullary and extramedullary lesions with only minimal activity detected (Figure 1). We stopped therapy on the patient’s request, and remission was kept for a total of more than 12 months. We summarized the treatment regimens and response in Table 1. Supplemental Figures S1 and S2 displayed the Ig A and free lambda light chain level in the entire course of the disease, respectively.

**Table 1.** Overview of treatment and response.

Line of therapy	Number of cycles	Therapy regimen and dosing	Best response
1	1	Cyclophosphamide 1500 mg/m^2^ on day 1; doxorubicin 15 mg/m^2^ on days 1–4; dexamethasone 40 mg on days 1–4; followed by stem cell mobilization	sCR
	2	High-dose melphalan (140 mg/m^2^) with autologous stem cell transplant	
	4	Consolidation with bortezomib 1.6 mg/m^2^ on days 1, 8, 15, 22; 1 cycle = 28 days	
2	17	Lenalidomide 25 mg on d1-21; dexamethasone 20 mg on days 1, 8, 15, 22; 1 cycle = 28 days	VGPR
	22	Lenalidomide 15 mg on d1-21; dexamethasone 20 mg on days 1, 8, 15, 22; 1 cycle = 28 days	
3	2	Bortezomib 1.3 mg/m^2^ on days 1, 4, 8, 11; dexamethasone 20 mg on days 1, 2, 4, 5, 8, 9, 11, 12; 1 cycle = 21 days	PD
4	6	Pomalidomide 4 mg on days 1–14; bortezomib 1 mg/m^2^ on days 1, 4, 8, 11; doxorubicin 9 mg/m^2^ on days 1–4; dexamethasone 20 mg on days 1, 2, 4, 5, 8, 9, 11, 12; 1 cycle = 21 days	VGPR
5	12	Carfilzomib 20/27 mg/m^2^ on days 1, 2, 8, 9, 15, 16; cyclophosphamide 300 mg/m^2^ on days 1, 8, 15; dexamethasone 20 mg on days 1, 2, 8, 9, 15, 16; 1 cycle = 28 days	VGPR
6	13	MOR202 16 mg/kg on days 1, 8, 15, 22; pomalidomide 4 mg on days 1–21; dexamethasone 40 mg on days 1, 8, 15, 22; 1 cycle = 28 days	VGPR
7	9	Pomalidomide 4 mg on days 1–14; bortezomib 1.3 mg/m^2^ on days 1, 4, 8, 11; doxorubicin 9 mg/m^2^ on days 1–4; dexamethasone 20 mg on days 0, 1, 2, 4, 5, 8, 9, 11, 12; daratumumab 16 mg/kg on days 0, 5; 1 cycle = 21 days	sCR MRD(–) at 10^−5^

MRD: minimal residual disease; PD: progressive disease; sCR: stringent complete remission; VGPR: very good partial remission.

## Conclusion

CD38 antibodies induce cell death in MM via various mechanisms, for example, complement-dependent cytotoxicity (CDC), antibody-dependent cellular cytotoxicity (ADCC), antibody-dependent cellular phagocytosis (ADCP), and immunomodulatory effects.^4^ To date, daratumumab, isatuximab, and MOR202 have shown promising anti-MM activity in diverse clinical trials.^7–9^ However, the majority of the initially responding patients progressed during therapy with CD38 antibodies.^10^ The management of these patients represents a new challenge in the clinical practice.

At the last relapse, our patient could not be treated with novel therapies within a clinical trial due to secondary malignancy, that is, invasive breast cancer. In this situation, German laws allow experimental treatment (“individueller Heilversuch”) and we started a multi-agent therapy with “Pom-PAD-Dara,” which resulted in sustained deep remission. Our case showed the synergistic effects of anti-MM agents, and in particular we provided the first evidence of successful CD38 antibody retreatment in patients with prior progression on CD38 targeted therapy. On the other hand, the effect of other anti-MM drugs such as pomalidomide, doxorubicin, and bortezomib, was exerted, and a good response could be obtained in addition to the effect of re-administration of CD38 antibody.

Decreased CD38 expression, overexpression of complement inhibitors (e.g. CD46, CD55, and CD59), acquired genetic or epigenetic alterations have been shown to be potential mechanisms of resistance to CD38 antibodies.^11–14^ Recently, it was reported that daratumumab refractory MM patients could respond to re-treatment with daratumumab.^15,16^ In our patient, the sustained deep remission to “Pom-PAD-Dara” after progression during MOR202-containing treatment was consistent with observations in previous reports, and underlined the feasibility of CD38 antibody re-treatment in RRMM patient. Other CD38 directed agents, such as isatuximab, are reported to inherit further cytotoxic mechanisms of action, in particular direct cell death induction, even in cells harboring *p53* mutations.^17,18^ This may suggest potential clinical value in CD38 retreatment strategies. However, clinical studies are needed to further evaluate these effects.

To the best of our knowledge, this is the first case of therapy response to daratumumab based combination in the direct line following to prior MOR202 containing therapy. Our case demonstrated the feasibility and efficacy of CD38 antibody retreatment using another agent in a heavily pretreated, penta-refractory patient. Clinical studies investigating CD38 retreatment strategies, either using the same or different CD38 targeting agents are urgently needed as a better understanding of the underlying resistance mechanisms.

## Supplemental Material

sj-pdf-1-iji-10.1177_2058738420980258 – Supplemental material for Sequential CD38 monoclonal antibody retreatment leads to deep remission in a patient with relapsed/refractory multiple myeloma Click here for additional data file. Supplemental material, sj-pdf-1-iji-10.1177_2058738420980258 for Sequential CD38 monoclonal antibody retreatment leads to deep remission in a patient with relapsed/refractory multiple myeloma by Maximilian Johannes Steinhardt, Xiang Zhou, Franziska Krummenast, Katharina Meckel, Katharina Nickel, David Böckle, Janin Messerschmidt, Sebastian Knorz, Alexander Dierks, Anke Heidemeier, Constantin Lapa, Hermann Einsele, Leo Rasche and Klaus Martin Kortüm in International Journal of Immunopathology and Pharmacology
