# Supplementary material for: Sequential CD38 monoclonal antibody retreatment leads to deep remission in a patient with relapsed/refractory multiple myeloma
Source: Int J Immunopathol Pharmacol. 2020 Dec 23;34:2058738420980258. doi: 10.1177/2058738420980258 (PMC7768838; doi:10.1177/2058738420980258)
Supplement: sj-pdf-1-iji-10.1177_2058738420980258 – Supplemental material for Sequential CD38 monoclonal antibody retreatment leads to deep remission in a patient with relapsed/refractory multiple myeloma [file sj-pdf-1-iji-10.1177_2058738420980258.pdf]

Supplemental material:

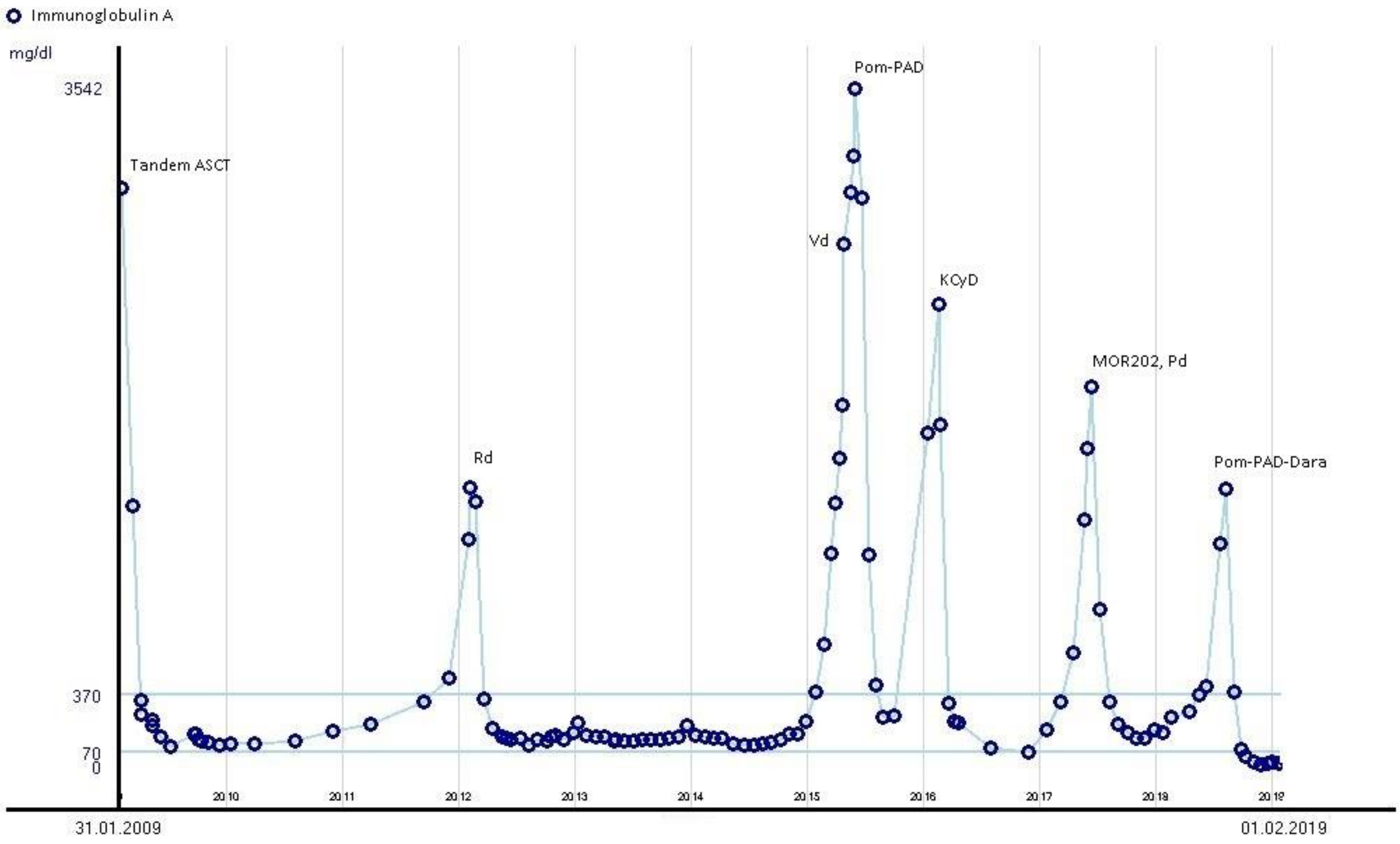

Figure S1: Immunoglobulin A (IgA) levels during the entire course of the disease. Therapy regimens were shown in the figure. Abbreviations: ASCT – autologous stem cell transplant; Rd – lenalidomide, dexamethasone; Vd – bortezomib, dexamethasone; Pom-PAD – pomalidomide, bortezomib, doxorubicin, dexamethasone; KCyD – carfilzomib, cyclophosphamide, dexamethasone; Pd – pomalidomide, dexamethasone; Pom-PAD-Dara – pomalidomide, bortezomib, doxorubicin, dexamethasone, daratumumab.

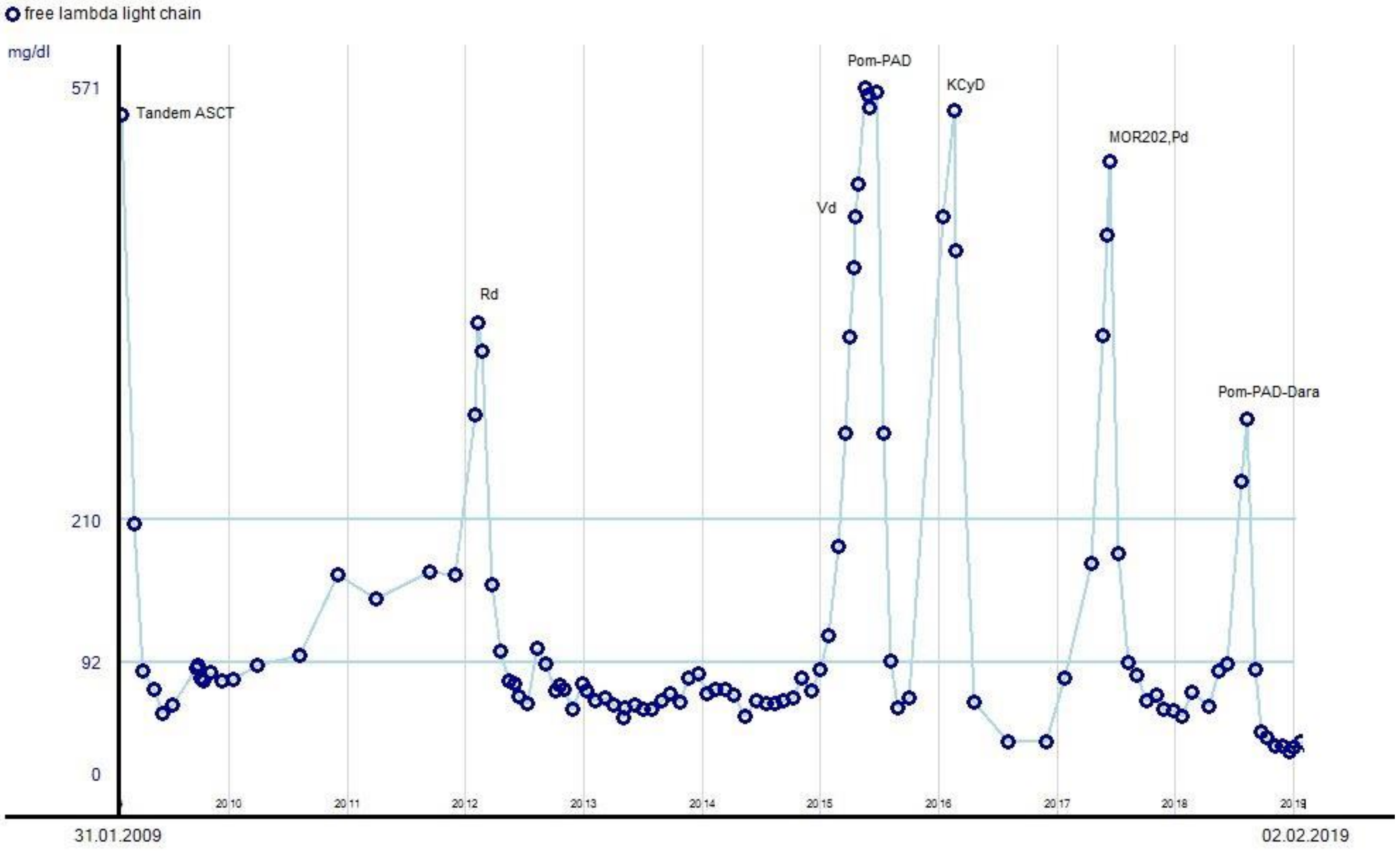

free lambda light chain
mg/dl
571
210
92
0
Tandem ASCT
Rd
Vd
Pom-PAD
KCyD
MOR202,Pd
Pom-PAD-Dara
2010
2011
2012
2013
2014
2015
2016
2017
2018
2019
31.01.2009
02.02.2019

Figure S2: Free lambda light chain levels during the entire course of the disease. Therapy regimens were shown in the figure. Abbreviations: ASCT – autologous stem cell transplant; Rd – lenalidomide, dexamethasone; Vd – bortezomib, dexamethasone; Pom-PAD – pomalidomide, bortezomib, doxorubicin, dexamethasone; KCyD – carfilzomib, cyclophosphamide, dexamethasone; Pd – pomalidomide, dexamethasone; Pom-PAD-Dara – pomalidomide, bortezomib, doxorubicin, dexamethasone, daratumumab.
